# The prone 12 o'clock position reduces ileal intubation time during colonoscopy compared to the left lateral 6 o'clock (standard) position

**DOI:** 10.1186/1471-230X-11-89

**Published:** 2011-08-04

**Authors:** Arjuna P De Silva, Ravindu S Kumarasena, Suramya D Perera Keragala, Udaya Kalubowila, Madunil Niriella, Anuradha S Dassanayake, Arunasalam Pathmeswaran, H Janaka de Silva

**Affiliations:** 1 Departments of Medicine, Faculty of Medicine, University of Kelaniya, Ragama, Sri Lanka; 2 North Colombo Teaching Hospital, Ragama, Sri Lanka; 3 Department of Pharmacology, Faculty of Medicine, University of Kelaniya, Ragama, Sri Lanka; 4 Department of Public Health, Faculty of Medicine, University of Kelaniya, Ragama, Sri Lanka

## Abstract

**Background:**

Ileal intubation is the gold standard for a complete colonoscopy. However, despite evidence of clinical benefit ileoscopy is not always attempted due to perceived technical difficulty. Our aim was to compare time taken for ileal intubation using a new position-the prone 12 o'clock position (PP) with the standard method (left lateral 6 o'clock position-LLP).

**Methods:**

We performed a pilot study using fluoroscopy to determine the best patient position for ileal intubation. This was the prone 12 o'clock position. Patients were colonoscoped in the left lateral position and then randomized to ileal intubation in the 6 o'clock position(LL) or the 12 o'clock (PP) position.

**Results:**

202 consecutive patients were referred for colonoscopy. Colonoscopy was performed on 150 patients [82 females, mean (SD) age 53 (16) years]. 75 patients were randomized for ileal intubation in the PP and 75 patients in the LLP. Overall, the ileum was successfully intubated in 145 (96%) patients [74 (98.7%) in the PP and71 (94.7%) in the LLP]. The median (Interquartile Range) ileal intubation time was 12 (10) seconds in the PP and 87 (82) seconds in the LLP (p < 0.0001; Mann-Whitney U test). The ileum was abnormal in 11 (7.5%) patients.

**Conclusions:**

During colonoscopy, the prone 12 o'clock position gives a more direct approach to the ileo-caecal valve and significantly reduces ileal intubation time.

**Trial registration:**

Trial registry: Sri Lanka Clinical Trial Registry

Clinical trial registry number: SLCTR/2009/002

## Background

Colonoscopy is a widely practiced procedure. Ileal intubation is widely regarded as the gold standard for evidence of complete colonoscopy [1]. However, this is not routinely attempted because of perceived technical difficulty, excess time thought to be added to the procedure or the low diagnostic yield that it was thought to provide [2]. However, there is mounting clinical evidence that ileoscopy is of clinical benefit [3]. It also important to remember that if ileoscopy is not routinely practiced, performing an ileoscopy may become difficult even when there is a definite clinical indication for doing so, such as, when Crohn's disease or ileal tuberculosis is suspected.

Currently the position employed to intubate ileum is with the patient in the left lateral position and entering the valve at the 6 o' clock position [4]. However, we have sometimes encountered difficulty when performing ileoscopy in this position leading to extra time being taken during busy endoscopy lists. During such difficult procedures we found that placing the patient in the prone position facilitated ileal intubation.

The aim of our study was to test the hypothesis that the prone position made ileal intubation easier and quicker than the standard position that is currently used-the left lateral position.

## Methods

**Figure 1:**
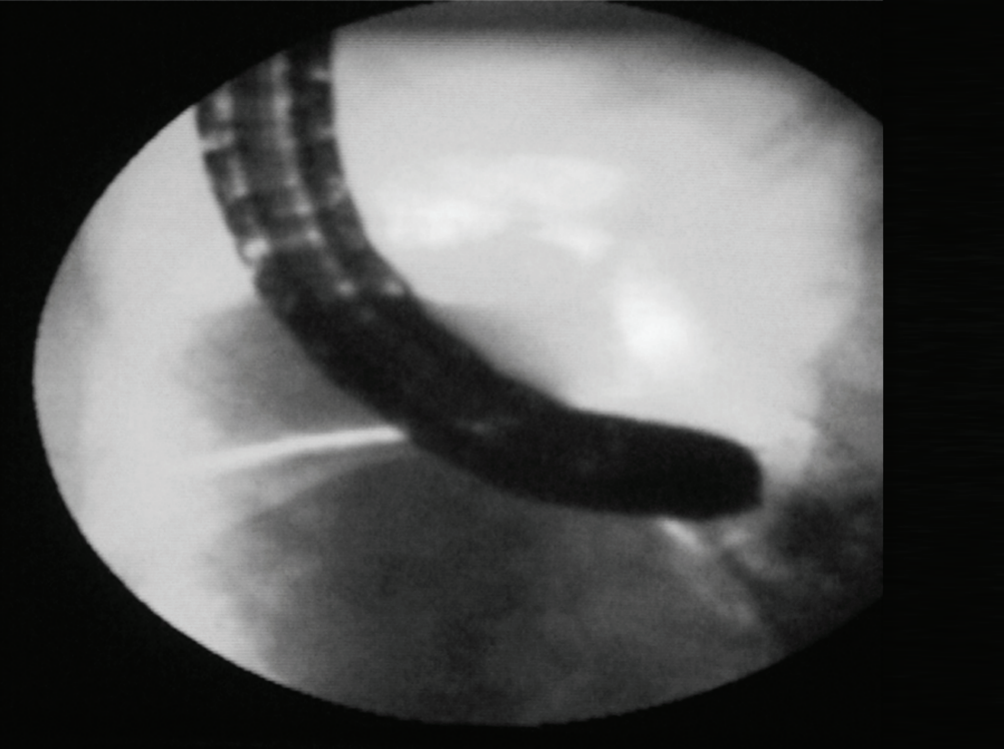
**The 12 o'clock position**.

**Figure 2:**
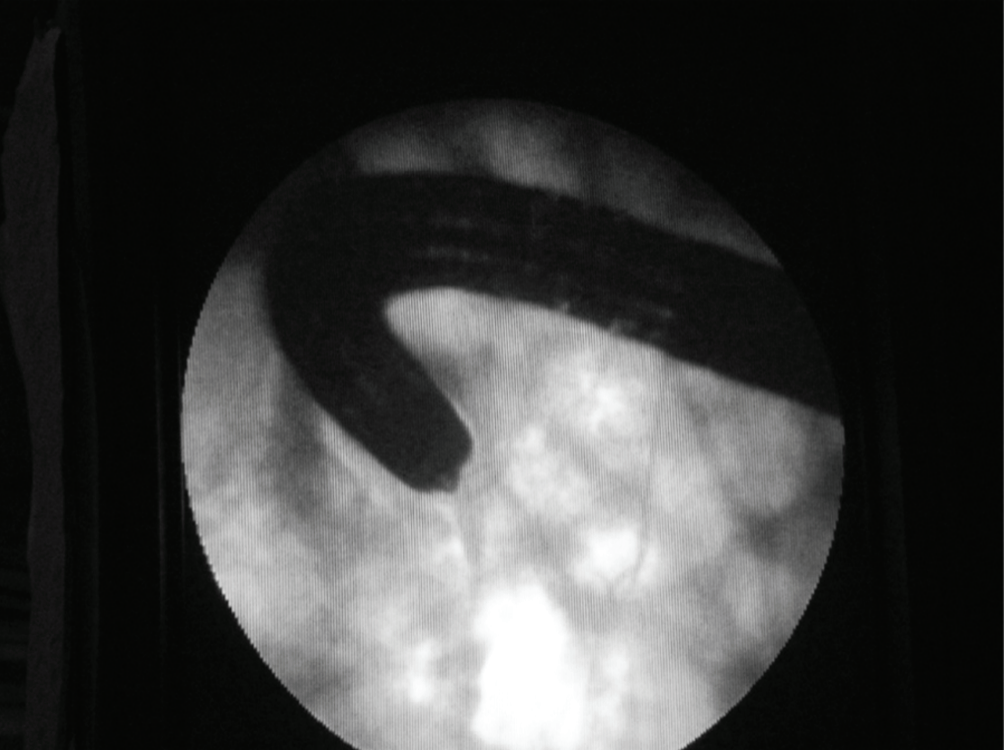
**The 6 o'clock position**.

We first performed a pilot study on ten patients undergoing routine colonoscopy using fluoroscopy to determine the best patient position for the most direct (end-on) approach to the ileo-caecal valve. Confirming our clinical impressions, the prone 12 o'clock position appeared to be the best position as this brought the tip of the colonoscope in line with the ileocaecal valve (Figure 1). This was unlike in the 6 o'clock position where the tip of the colonoscope was curved and not in the same axis (Figure 2).

We then randomized consecutive patients referred for colonoscopy to our unit between February2009 and Jan 2010 using computer generated random numbers. Patients aged between 18-80 years and who were not pregnant were recruited after obtaining their written informed consent. They were then randomized to undergo ileoscopy either in the standard position or the prone 12 o'clock position.

All patients were given four packets of polyethylene glycol (PEG) for bowel cleansing prior to colonoscopy. All patients received pre-medication with midazolam 2.5 mg i.v. and pethidine 25 mg i.v. All patients had pulse oximetry monitoring during the procedure. None of the patients were given hyoscine-n-butyl bromide. The colonoscopes used were Olympus GF Q145L models.

All colonoscopies were performed by experienced endoscopists (MAN and KVUK). The procedures were started in the left lateral position and the position changed after reaching the caecum, according to randomization. After the ileo-caecal valve was identified, ileal intubation time was standardized, and defined as the time taken for the tip of the colonoscope to be maneuvered from the mid-point of the caecum to entering the terminal ileum. This was timed by an independent observer (RSK).

### Ethics

Ethical clearance for the study was obtained from the Ethics Committee of the Faculty of Medicine, University of Kelaniya, Sri Lanka. Informed written consent was obtained from all patients.

### Statistics

Sample size calculation was done on an assumption of 75% v 95% success at ileal intubation with the PP comp, and at 90% power this required a sample of 150 patients. The data was compared using Chi squared test and the statistical difference between the two groups will compared using the program SPSS 16.

## Results

**Figure 3:**
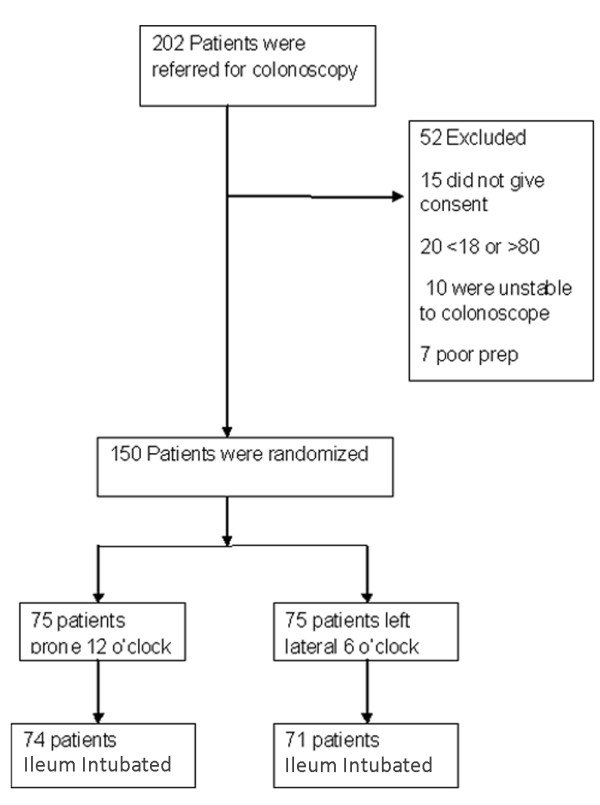
**Trial profile**.

Two hundred and two consecutive patients were referred for Colonoscopy during the study period (Figure 3). Colonoscopy was performed on 150 patients [82 females, mean (SD) age 53 (16) years]. 75 patients were randomized for ileal intubation in the PP(prone position) and 75 patients in the LLP(left lateral position). The two groups were comparable for age, sex, indication for colonoscopy (Table 1). Overall, the ileum was successfully intubated in 145 (96%) patients [74 (98.7%) in the PP and71 (94.7%) in the LLP] (Table 2). The median (Interquartile Range) ileal intubation time was12 (10) seconds in the PP and 87(82) seconds in the LLP (p < 0.0001; Mann-Whitney U test). The ileum was abnormal in 11 (7.5%) patients: 6 in the PP group and 5 in the LLP group.

**Table 1:** Demography and Indication for Colonoscopy

Variables	Prone 12 (n = 75)	Left lateral (n = 75)
Mean age(Range) years	50(18-80)	55(18-80)

Males:Females	40:35	42:33

Diarrhoea	8	5

Constipation	8	12

Altered bowel habits	19	18

Abdominal pain	16	12

Iron deficiency Anaemia	9	10

Per rectal bleeding	3	6

IBD†	6	8

Carcinoma of unknown primary	3	1

Loss of weight or/and Loss of appetite	3	3

†-Inflammatory Bowel Disease

**Table 2:** Ileal Intubation Time and Finding

Variables	Prone 12 (n = 75)	Left lateral (n = 75)	P Value
Median (inter quartile range) time taken for ileal intubation (seconds)	12 (5-15)	87 (18-100)	0.0001 *

Median (inter quartile range) time taken for colonoscopy (seconds)	910 (670-1420)	977 (680-1400)	0.89*

Ileal finding Number of patients with ileitis	6	5	0.75**

*Based on Mann-Whitney Test

**Based on Pearson's chi-square Test

## Discussion

We have shown that during colonoscopy, the prone 12 o'clock position gives a more direct approach to the ileo-caecal valve and, although the ileum was intubated in more than 90% of cases in both positions, significantly reduces ileal intubation time when compared to the standard left lateral 6 o'clock position. The reason for this is that in the prone 12 o'clock position, the axis of the tip of the colonoscope is the same as the ileocaecal valve (as clearly demonstrated during fluoroscopy). This makes entry into the ileocaecal valve much easier. The ileal abnormality rate was similar in both groups, and would therefore have not confounded our results.

There are a few aspects of our study that warrant discussion. We did not assess other positions for ease of ileal intubation. However, we used the best position established by other studies and what is generally accepted as the best position (6 o'clock position) versus what we empirically thought and was also supported by fluoroscopy as was the best position (12 o'clock). Although our analysis did not take into account possible confounding factors such as the endoscopist, nurses and bowel preparation, we attempted to minimize these by having the same nursing team, two colonoscopists and using standard bowel preparation for all our patients during the study. The argument regarding the value of ileal intubation itself also warrants comment. Some studies have shown that only very few (1%) patients who undergo ileoscopy have abnormalities in the ileum [5]. However, other studies including our own data show much higher rates of detection of ileal pathology [3,6].

Although several previous studies have reported on the time taken for ileal intubation, such timings have not been standardized [6]. This has resulted in varying definitions of ileal intubation times which are not comparable, and the times reported range from seconds in some studies to more than ten minutes in some [7]. While no studies have clearly stated how to define ileal intubation time, it is assumed to be the time taken to maneuver the endoscope from the tip of the valve into the terminal ileum [4,8]. We felt that this does not give a true reflection of the difficulty of the procedure. We, therefore, defined it as the time taken for the tip of the colonoscope to be maneuvered from the mid-point of the caecum to entering the terminal ileum. Furthermore, we did not design our trial as a cross over study because once the ileum is intubated, the valve becomes patulous making the second intubation is easier [4]. Although the time difference between the two methods may not have much clinical significance (12 seconds vs 87 seconds) it may be significant for training purposes.

## Conclusion

In conclusion, we recommend the prone 12 o'clock position for ileal intubation during colonoscopy as it is easier and takes less time.

## Competing interests

The authors declare that they have no competing interests.

## Authors' contributions

APDS and HJdS were involved in conceptualizing and writing the study. KSK, MN and UK, were involved in colonoscopy. ASD was involved in the patient care. BSDPK was involved in data gathering. AP was involved in the statistical analysis. All authors read the manuscript and were involved in editing the final copy.

## Pre-publication history

The pre-publication history for this paper can be accessed here:

http://www.biomedcentral.com/1471-230X/11/89/prepub
